# The Effects of Target Location Upon Throwing Velocity and Accuracy in Experienced Female Handball Players

**DOI:** 10.3389/fpsyg.2020.02006

**Published:** 2020-08-07

**Authors:** Roland van den Tillaar

**Affiliations:** Department of Sports Science and Physical Education, Nord University, Levanger, Norway

**Keywords:** overarm throwing, coordination, ball velocity, accuracy, Fitts’ law, speed accuracy trade-off, motor control

## Abstract

The purpose of this study was to investigate the effect on throwing performance (velocity and accuracy) of experienced female handball players when throwing at four different targets in a handball goal. Thirteen experienced female handball players (age 18.2 ± 1.7 years, height 1.7 ± 0.10 m, mass 68.1 ± 19.6 kg, and training experience 9.5 ± 3.7 years) performed 10 throws from a 7 m distance at each corner of the handball goal with maximal effort. Maximal ball velocity was recorded with a radar gun together with mean radial error, centroid error, and bivariate variable error, as measurements of accuracy. The main findings were that the ball velocity was higher when throwing at targets at the ipsilateral side, compared with the contralateral side, while throwing consistency (bivariate variable error) decreased when throwing at the contralateral side upper corner. No velocity-accuracy trade-off was found between the four targets. Based upon the findings, it is suggested that players throw to the (upper) ipsilateral side of the goal when performing a penalty throw, dependent on the goalkeepers’ position, since the ball velocity is the highest here, without losing accuracy. This gives the goalkeeper less time to react and stop the ball, thereby giving the player the highest chance of scoring.

## Introduction

Team handball is an Olympic team sport in which two teams of seven players each, compete against each other. The most important task for both teams is to score more goals than the opponent. To score goals, accuracy and velocity are the two main factors. There are several possible strategies of performing a goal-directed throw: simply throwing as fast as possible without any intent to aim accurately, trying to surprise the opponent/goalkeeper by the velocity of the throw (goalkeeper independent strategies) or by deceptive actions when performing a throw or throwing as accurately as possible, and trying to keep the ball out of reach of the opponent (goalkeeper dependent strategies; van der Kamp, 2006).

Different trade-offs between velocity and accuracy are reported in the literature based on different theoretical principles that apply to different movements (Fitts, 1954; Sherwood and Schmidt, 1980; Plamondon and Alimi, 1997). In these studies, it was found that velocity and accuracy were influenced in an inverse manner. Thus, when aiming for velocity the accuracy decreases and when aiming for accuracy the velocity decreases. This relationship was also found in earlier studies in handball throwing (Indermill and Husak, 1984; Etnyre, 1998). However, later studies showed that this happens only in part (van den Tillaar and Ettema, 2003a,b, 2006, 2009a); throwing velocity decreases when accuracy was more important, but the accuracy of the performance was not better when focusing upon accuracy; thereby these findings did not follow the traditional speed-accuracy trade-off, also called Fitts’ law (Fitts, 1954).

However, in all of these previous studies in handball, the target was straight forward, which does not have much external validity in the sport, because this is where the goalkeeper stands. Rivilla-Garcia et al. (2011) found that throwing velocity was already decreased when a goalkeeper was in the goal compared to throws without an opponent. Hence, it seems reasonable to suggest that throwing to the left and right, upper and lower corners of the goal is initiated by different control and movement strategies than straight forward throws and thereby perhaps use different motor program schemas (Schmidt, 1982). Detailed knowledge about this may help to clarify the underlying mechanisms of the speed-accuracy trade-off (Fitts, 1954; Plamondon and Alimi, 1997). Additionally, the findings may have some practical implications regarding which corner of the goal you should throw to, in order to have the largest chance of succeeding (highest throwing velocity and/or accuracy) when the strategy of the player is goalkeeper independent. In the keeper-independent strategy, the shooter selects a target location in advance and disregards the goalkeeper’s actions (van der Kamp, 2006), in which velocity is the main aim and accuracy is the secondary aim (van den Tillaar and Ettema, 2003a, 2006, 2009a).

Therefore, the purpose of this study was to investigate the effects of target location upon throwing performance (velocity and accuracy) in experienced female handball throwers in 7 m throws. It was hypothesized that maximal ball velocity to the contralateral side of the throwing arm is higher than on the ipsilateral side due to the possible use of the longitudinal rotation of the pelvis and trunk (Wagner et al., 2010, 2011), causing a longer working trajectory, as found in soccer kicking (van den Tillaar and Fuglstad, 2016). In addition, it can be hypothesized that when the throwing velocity is higher, the accuracy of throwing decreases. Thus, the target in which the ball velocity is the highest will have the lowest accuracy, following Fitts’ law (Fitts, 1954). Thereby, it is expected that throwing to the contralateral side would result in higher velocity but with lower accuracy then throws to the ipsilateral side.

## Materials and Methods

I used a repeated-measures design to investigate the effects of target location upon throwing performance (velocity and accuracy). Each subject performed 10 throws at each target in a random order.

### Participants

Thirteen female team handball players (age 18.2 ± 1.7 years, height 1.7 ± 0.10 m, body mass 68.1 ± 9.6 kg, and training experience 9.5 ± 3.7 years), playing in the highest Norwegian national competition, volunteered for the study. Testing was conducted in the middle of the handball season (January–February) always between 10 AM and 5 PM. The participants were fully informed about the protocol before participating in this study. Informed consent was obtained prior to all testing from all participants and their parents, with the approval of the Norwegian Centre for Research Data and a further approval by an Ethics Committee was not required as per applicable institutional and national guidelines and regulations.

### Procedure

**Figure 1:**
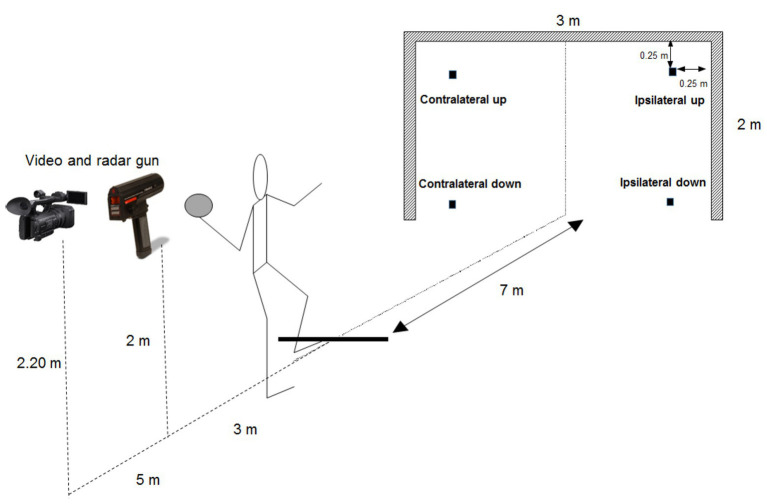
Experimental setup with the different targets and distances.

After a general warm-up of 15 min, which included some jogging and warming up the shoulder and throwing arm, throwing performance was tested in a 7 m throw situation as this is a penalty throw regularly performed in handball. The subjects performed a standing throw, which means keeping the front foot on the floor the whole-time during throwing. The participants started by holding the ball with both hands in front of them. The subjects were instructed to throw as fast as possible and try to hit the target (van den Tillaar and Ettema, 2003b) from 7 m distance with a regular ball (0.35 kg), aiming at one of the four targets located 0.25 m from each corner (up and down) of the standard handball goal (2 × 3 m), which was drawn upon a wall (Figure 1). All subjects were right-handed except one for whom everything was mirrored. Each subject was instructed to throw 10 times at each of the four target locations, resulting in 40 throws per subject. The different target locations were given in a random order to avoid fatigue, learning or any other time-related effects, which might affect the results in a systematic way. The random order was based on a random number generator. The subjects had approximately a 1-min rest between each throw.

### Measurements

Maximal ball velocity was measured using a Doppler radar gun (Stalker ATS II, Applied Concepts Inc., Plano, Texas) with ±0.028 m/s accuracy within a field of 10 degrees from the gun. The radar gun was located 2 m behind the participant, at throwing height, during the throw.

**Figure 2:**
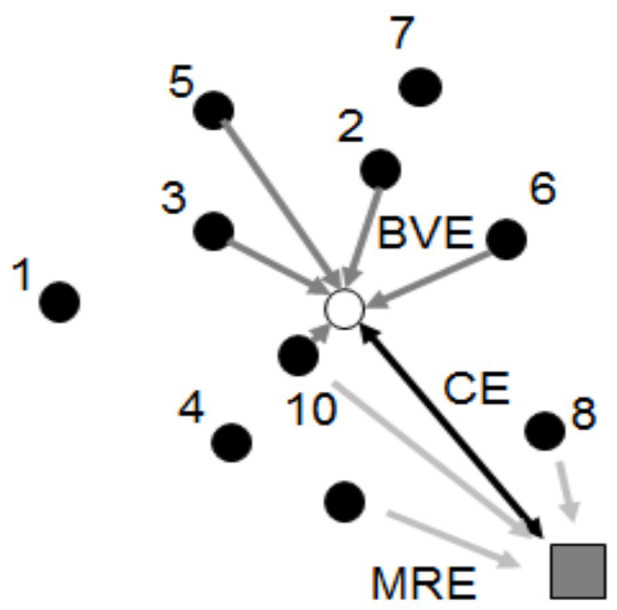
Accuracy measurements for each target. Mean radial error (MRE) was measured as the average of absolute distance to the center of the target. The subject’s own midpoint is measured as the average hit location over all trials per target per subject, whereby the centroid error (CE) is the absolute distance of the subject’s own midpoint to absolute target midpoint. Bivariate variable error (BVE), also referred to as consistency, is the average of the absolute distance to the subject’s own midpoint.

Throwing accuracy was measured (50 Hz) with a video camera (Sony HDR FX 1000, Tokyo, Japan) at a distance of 15 m from the goal. The camera was placed such that the subject did not obstruct the visual field of the camera toward the goal (Figure 1). The *x* and *y* positions of the center of the ball at the moment that the ball hit the wall (goal) from the center of the target location aim were measured with a ruler with an accuracy in mm, when the video camera was connected to a 0.6 by 1.0 m flat screen. The 2 by 3 m goal was used as a calibration frame. Accuracy was measured as mean radial error: the average of the absolute distance to the center of the target; bivariate variable error, also called consistency: the average of the absolute distance to the subject’s own midpoint; and centroid error, also called bias: the absolute distance of a subject’s midpoint to the absolute midpoint (Figure 2), as described by van den Tillaar and Ettema (2003b).

### Statistics

To compare the ball velocity and accuracy, a one-way ANOVA with repeated measures (four different target locations) was used with Holm-Bonferroni *post hoc* tests. When the assumption of sphericity was violated, the Greenhouse-Geisser adjustments of *p* were reported. The significance level was set at *p* ≤ 0.05. Statistical analysis was performed in SPSS version 24.0 (SPSS, Inc., Chicago, IL). All results are presented as mean ± standard deviations unless otherwise stated. Effect size was evaluated with *η*^2^ (eta partial squared), where 0.01 < *η*^2^ < 0.06 constitutes a small effect, 0.06 < *η*^2^ < 0.14 constitutes a medium effect, and *η*^2^ > 0.14 constitutes a large effect (Cohen, 1988).

## Results

**Figure 3:**
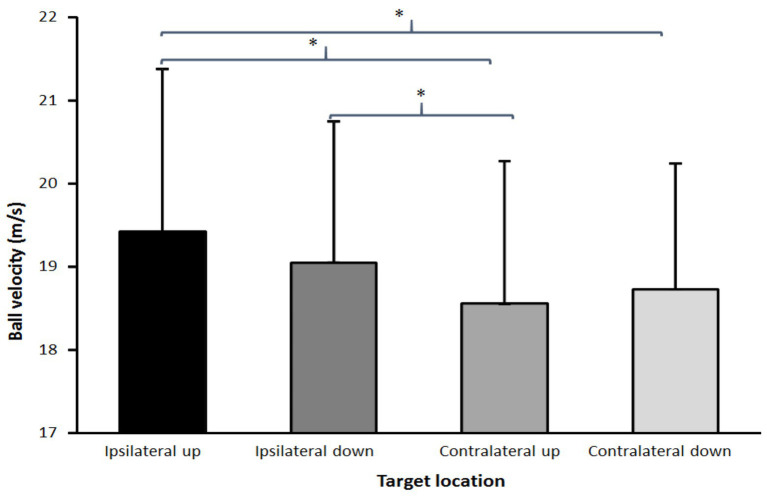
Average ball velocity for each target location in the goal from each target of the goal. ^*^indicates a significant difference in ball velocity between these two target locations on a *p* < 0.05 level.

**Figure 4:**
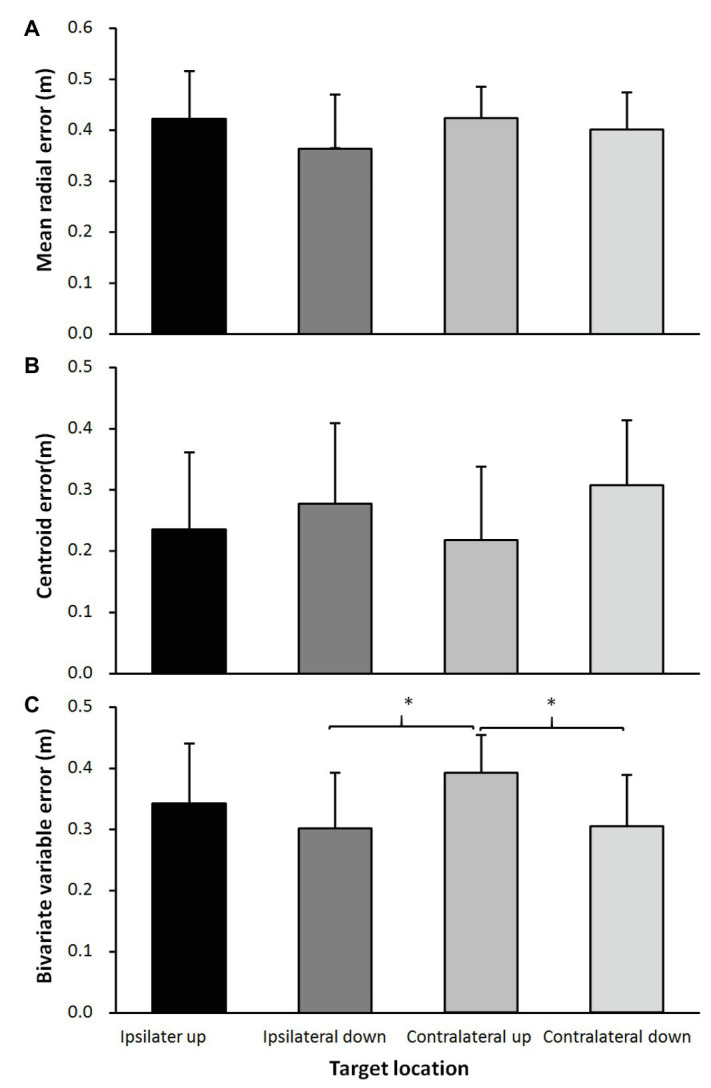
**(A)** MRE, **(B)** CE, and **(C)** BVE averaged over all participants for each target location. ^*^indicates a significant difference in ball velocity between these two target locations on a *p* < 0.05 level.

Maximal ball velocity was significantly affected by the target location (*F* = 11.4, *p* < 0.001, *η*^2^ = 0.49; Figure 3). *Post hoc* comparisons showed that the ball velocity was significantly higher when throwing at the upper ipsilateral side compared with both target locations on the contralateral side. In addition, the ball velocity was higher when throwing at the lower ipsilateral side corner compared to the upper contralateral side corner (Figure 3). Of the different accuracy measurements, only the bivariate variable error (*F* = 3.8, *p* = 0.017, *η*^2^ = 0.24; Figure 4C) was significantly affected by the target location, while no significant effects found for the centroid error (*F* = 1.4, *p* = 0.254, *η*^2^ = 0.11; Figure 4B), and the mean radial error (*F* = 1.8, *p* = 0.195, *η*^2^ = 0.13; Figure 4A). *Post hoc* comparisons showed that the bivariate variable error in the upper contralateral corner was significantly higher than in the two low corners in the goal (Figure 4C).

## Discussion

In this study, the effect of target location upon throwing performance (velocity and accuracy) in experienced female handball players was examined. The main findings were that the ball velocity was higher when throwing at targets at the ipsilateral side, compared with the contralateral side (Figure 3), while throwing consistency (bivariate variable error) decreased when throwing at a target at the upper corner of the contralateral side (Figure 4).

The findings on maximal ball velocity were opposite from the hypothesis, which was postulated. Higher ball velocity was expected at the contralateral side due to the possible use of the rotation around the longitudinal axis of the pelvis and trunk, as found in soccer kicking (van den Tillaar and Fuglstad, 2016). A possible discrepancy with the findings on soccer kicking is that, in overarm throwing, the main contributors of the maximal ball velocity are the internal shoulder rotation and elbow extension movements (van den Tillaar and Ettema, 2007). These two movements are forward-orientated, and a combination of these two movements will result in the ball traveling forward and to the ipsilateral side. Thereby, to shoot to the contralateral side, pelvis and/or trunk rotation should occur in order to aim to that side. The maximal pelvis (0.13–0.17 before ball release) and trunk rotation (0.06–0.08 before ball release) movements occur early in the throw, during the acceleration phase (Fradet et al., 2004; van den Tillaar and Ettema, 2007, 2009b; Wagner et al., 2010, 2011; van den Tillaar et al., 2013). These movements could contribute just a little to the maximal ball velocity (Wagner et al., 2010, 2011) due to the early occurrence of these movements compared to the maximal internal shoulder rotation and maximal elbow extension movements, which occur around ball release (±0.01 s before and after ball release). Since the timing of the maximal pelvis and trunk rotations occurs so early and timing is crucial for accurate throwing, it is probable that small inaccuracies in timing of these two movements cause differences in accuracy, as observed when throwing at the upper corner of the contralateral side (Figure 4). The consistency (bivariate variable error) is less here than at the targets in the two lower corners.

No other significant differences in hitting accuracy (mean radial error and centroid error) were found between any of the targets, indicating that accuracy does not change much when aiming for these targets. The average accuracy was similar to previous studies in which players had to throw straight forward (van den Tillaar and Ettema, 2003a,b, 2006, 2009a), indicating that throwing at a corner in the goal does not decrease hitting accuracy compared with throwing straight forward.

When the main attention is upon throwing as fast as possible and the secondary aim is trying to hit the target in one of the four corners of the goal, no velocity-accuracy trade-off occurs. This is in agreement with the findings of previous studies (van den Tillaar and Ettema, 2003a, 2006, 2009a) in which instructions with different priorities (velocity and/or accuracy) did not change accuracy, indicating that handball players do not follow Fitts’ law (Fitts, 1954), whereas in dart throwing (van den Tillaar and Aune, 2019) and soccer kicking (van den Tillaar and Ulvik, 2014; van den Tillaar and Fuglstad, 2016), a velocity-accuracy trade-off was found. Reason for this could be that in handball throwing with different priorities (velocity and/or accuracy) occurs at velocities of more than 85% of maximal ball velocity, in which execution force-variability decreases (Sherwood and Schmidt, 1980; Schmidt and Lee, 1999). Thereby, fast and discrete movements like overarm throwing become more consistent when performed with maximal or near maximal intensity. In dart throwing and soccer kicking studies by van den Tillaar and colleagues (van den Tillaar and Ulvik, 2014; van den Tillaar and Fuglstad, 2016; van den Tillaar and Aune, 2019), the velocity decreased to under 80% of maximum when accuracy was prioritized and, thereby, according to Schmidt and Lee (1999), force-variability increases which, again, increases inaccuracy. In the present study, no prioritizing was necessary; thereby, the lowest velocity (contralateral upper corner) was still 95% of maximal ball velocity compared with the ipsilateral upper corner, which caused no major accuracy changes to occur (Figure 4).

However, the present study has some limitations. Firstly, only 7 m throws were measured, which does not have much ecological validity during handball matches (except during penalty throws) since players in matches mostly throw from 6 to 10 m distance at the goal with a goalkeeper and defenders in between with a preliminary run up and/or jump (Michalsik et al., 2015). In addition, only women were measured. Men, in general, throw faster (van den Tillaar and Cabri, 2012) and perhaps, thereby, have a different velocity-accuracy profile than women. The experience level of the players was high but with higher level (international) or lower level players the findings could be different, which must be investigated in new studies before we can generalize the findings to larger populations. Furthermore, no 3D kinematics were conducted to investigate where, in the throwing movement, changes occur that cause these differences in velocity and accuracy. These measurements could give more information about movement control and performance determinants in throwing. Future studies should include 3D measurements, with men, from different levels and performed with a similar set up of targets in different corners, but from different player positions and with run up and/or jump to establish more detailed information about these variables.

## Conclusions

In summary, it can be stated that throwing at the different corners of a handball goal results in faster throws to the ipsilateral side than the contralateral side, while consistency (bivariate variable error) only decreases when throwing to the contralateral upper corner with experienced female handball players. Thereby, the study does not follow Fitts’ law (Fitts, 1954) that accuracy decreases when velocity increases and vice versa. As a result of the present study, it is suggested to players who want to use a goalkeeper independent throwing strategy, when performing a penalty throw, they throw to the (upper) ipsilateral side of the goal, since the ball velocity is the highest here, without losing accuracy. This gives the goalkeeper less time to react and stop the ball; thereby, the chance of scoring is the highest for the player.

## Data Availability Statement

The raw data supporting the conclusions of this article will be made available by the authors, without undue reservation.

## Ethics Statement

The studies involving human participants were reviewed and approved by Norwegian Centre for Research Data (project number: 42440). The participants provided their written informed consent to participate in this study.

## Author Contributions

The author did all the work in collaboration with a student who did the data collection.

### Conflict of Interest

The author declares that the research was conducted in the absence of any commercial or financial relationships that could be construed as a potential conflict of interest.
